# Supplementary material for: The origin, evolution and functional divergence of HOOKLESS1 in plants
Source: Commun Biol. 2023 Apr 26;6:460. doi: 10.1038/s42003-023-04849-4 (PMC10133230; doi:10.1038/s42003-023-04849-4)
Supplement: Supplementary file 2 — Supplementary Figures [file 42003_2023_4849_MOESM2_ESM.pdf]

Supplementary Figures

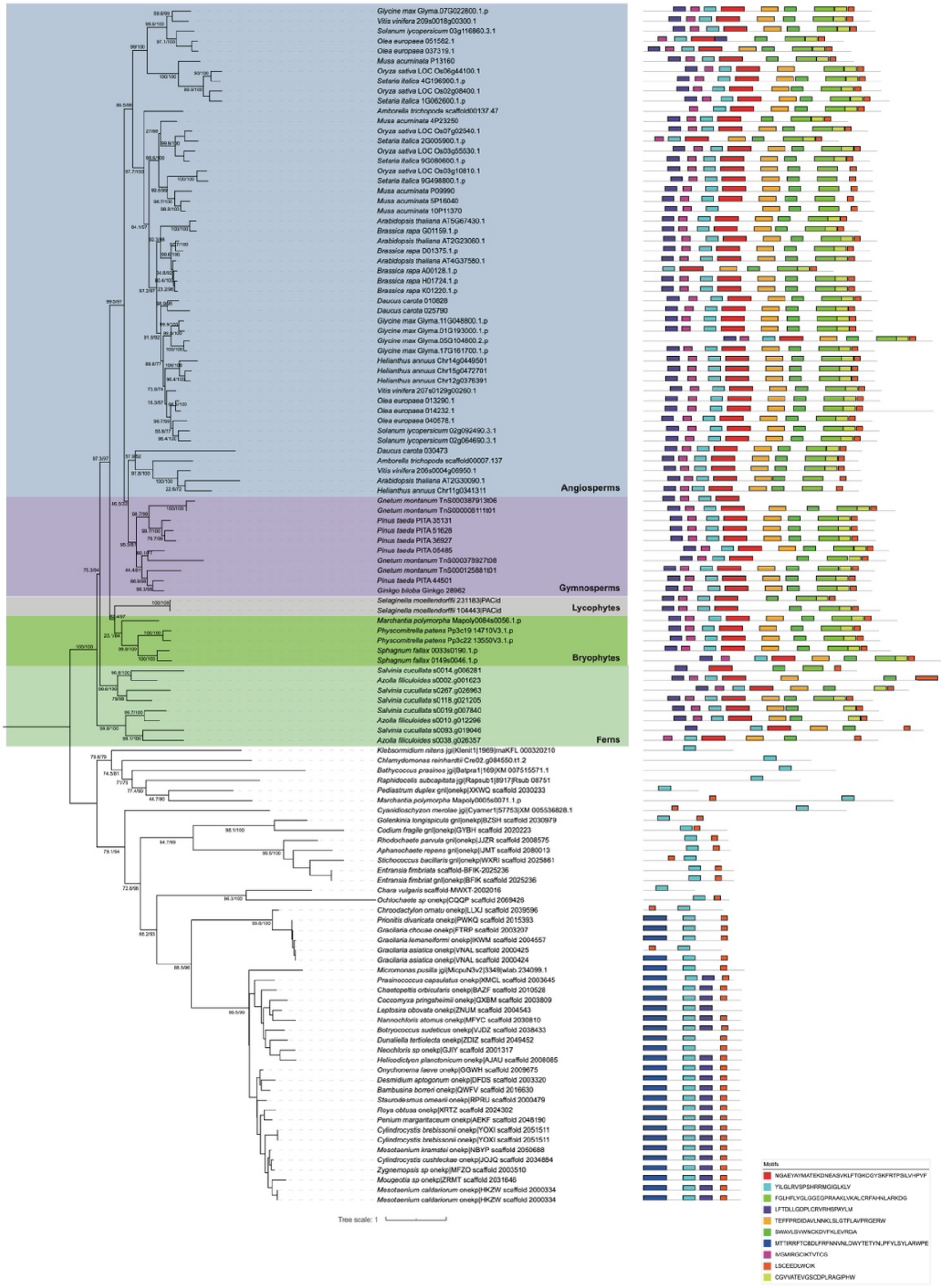

Supplementary Figure 1: **Phylogenetic tree and motifs comparison of plant HLS1 homologs.**

Nodal support values are estimated by SH-aLRT test (SH) and ultrafast bootstrap (UFBS) in IQ-TREE2. The predicted conserved motifs were mapped near each branch.

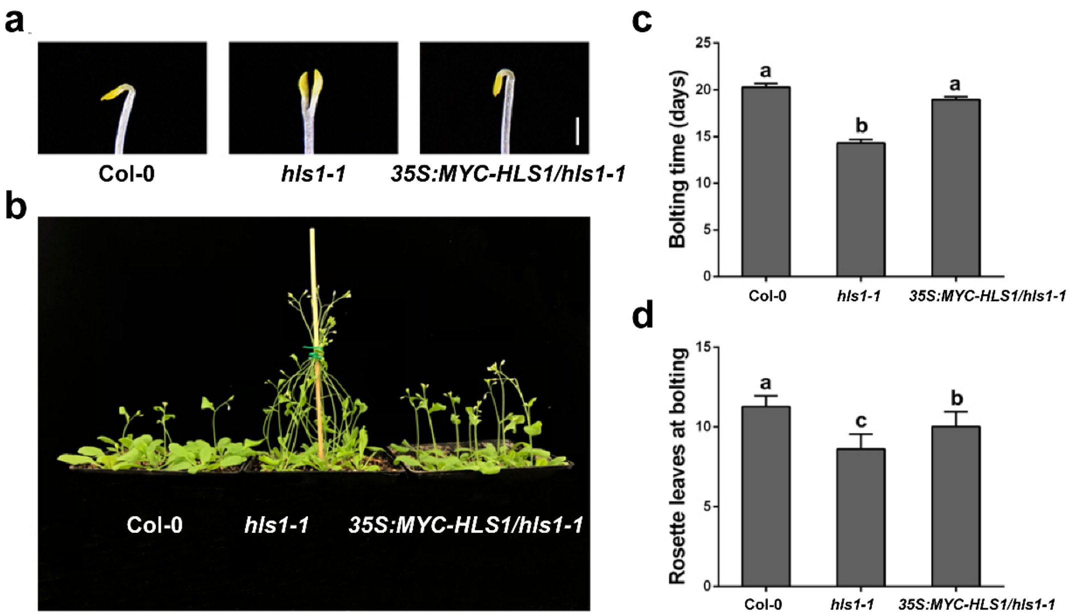

Supplementary Figure 2: **Flowering phenotypes in complementation lines**

(a) Representative images showing hook angle phenotypes in 4-day-old etiolated seedlings grown on MS medium.

(b) Images show representative flowering phenotypes of 27-day-old plants grown under long day (16 hr light/8 hr dark) condition.

(c-d) The days to flowering (bolting time) (c) and the rosette leaf numbers at bolting (d) were shown in charts. Different letters indicated significantly different values ($P < 0.05$, ANOVA followed by Tukey's test, data are means ± SD, $n \geq 30$).

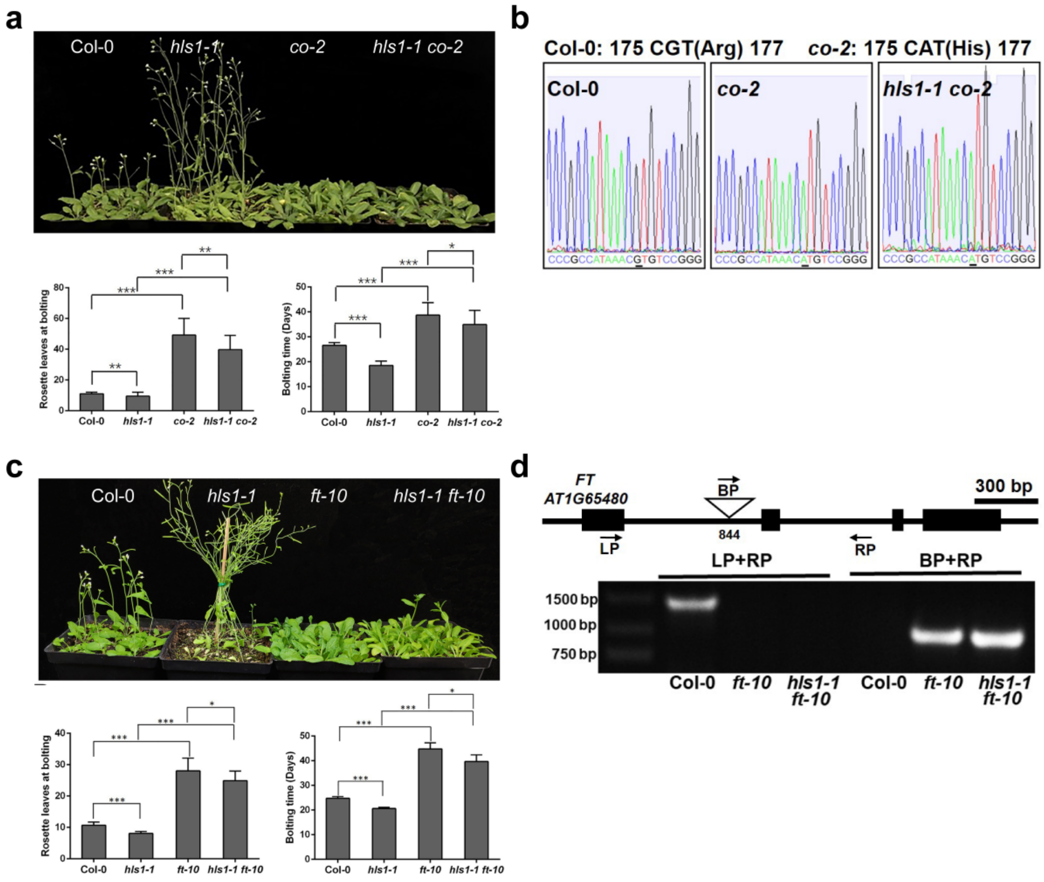

Supplementary Figure 3: **Genotyping of *hls1 co* or *hls1 ft* double mutants**

(a) Flowering phenotypes of *hls1 co* double mutants. Representative image showing 30-dayold plants grown under LD conditions. Quantitative results showing the flowering phenotypes of plants grown under LD conditions (Data are means ± SD; $n \geq 15$, ****P* < 0.001, t***P* < 0.01, **P* < 0.05 based on Student's *t* test).

(b) To characterize the homozygous *hls1-1 co-2* double mutants, F2 populations from the genetic cross between *hls1-1* and *co-2* were firstly screened in darkness to select *hls1-1* like lines. Then these lines were individually genotyped through PCR based sequencing to identify the *co-2* mutations (CGT to CAT change in the coding region). Representative Sanger-sequencing results were shown.

(c) Flowering phenotypes of *hls1 ft* double mutants. Representative image showing 33-day-old plants grown under LD conditions. Quantitative results showing flowering phenotypes of plants grown under LD conditions (Data are means ± SD; $n \geq 40$, ****P* < 0.001,***P* < 0.01, **P* < 0.05

based on Student's *t* test).

(d) To characterize the homozygous *hls1-1 ft-10* double mutants, F2 populations from the genetic cross between *hls1-1* and *ft-10* were firstly screened in darkness to select *hls1-1* like lines. Then these lines were individually genotyped through PCR based genotyping to identify the *ft-10* mutations (T-DNA insertion in intron region, depicted as a triangle in the cartoon). Representative genotyping results were shown.

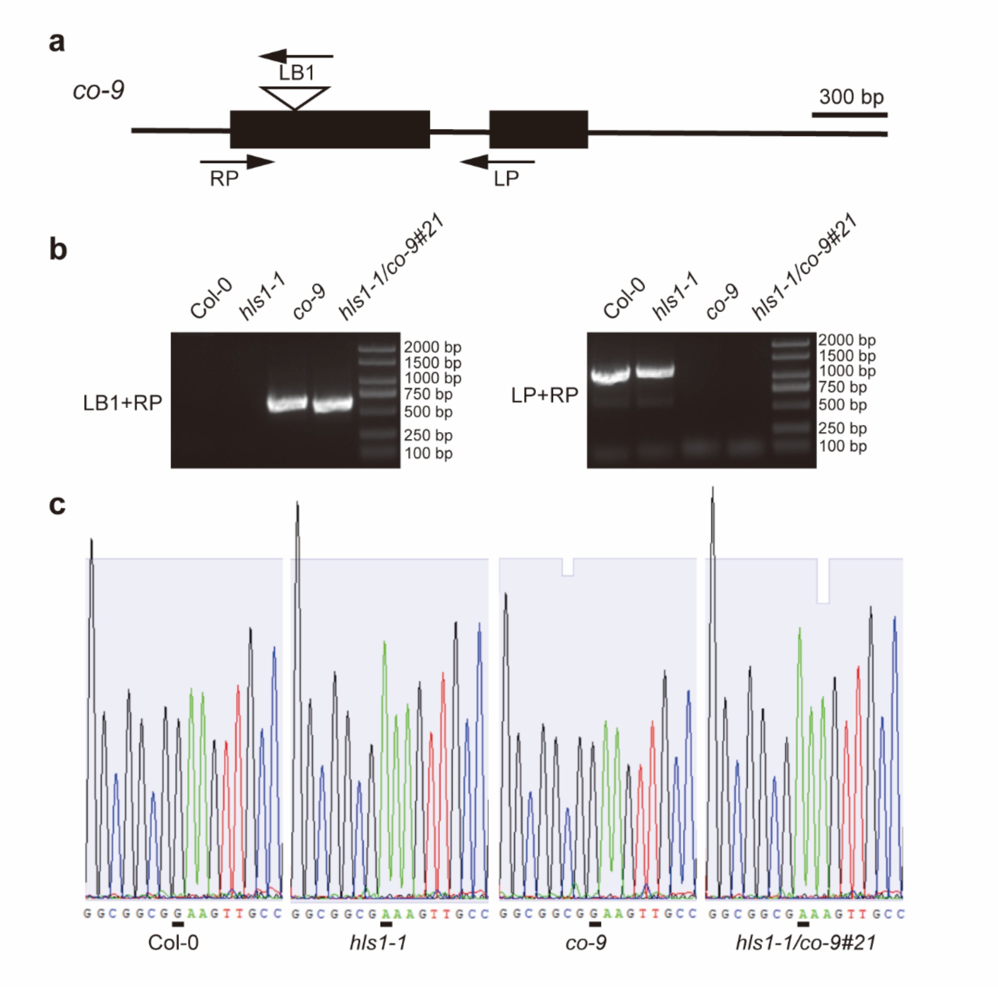

Supplementary Figure 4: **Genotyping of *hls1 co-9* mutants**

(a) Schematic illustration shows the T-DNA insertion in the first exon of *CO*. Black triangle represents the T-DNA insertion site.

(b) PCR based genotyping to identify the *co-9* mutations in the indicated F3 populations.

(c) Sanger-sequencing results in the indicate F3 populations to identify the homozygous *hls1-1* loci.

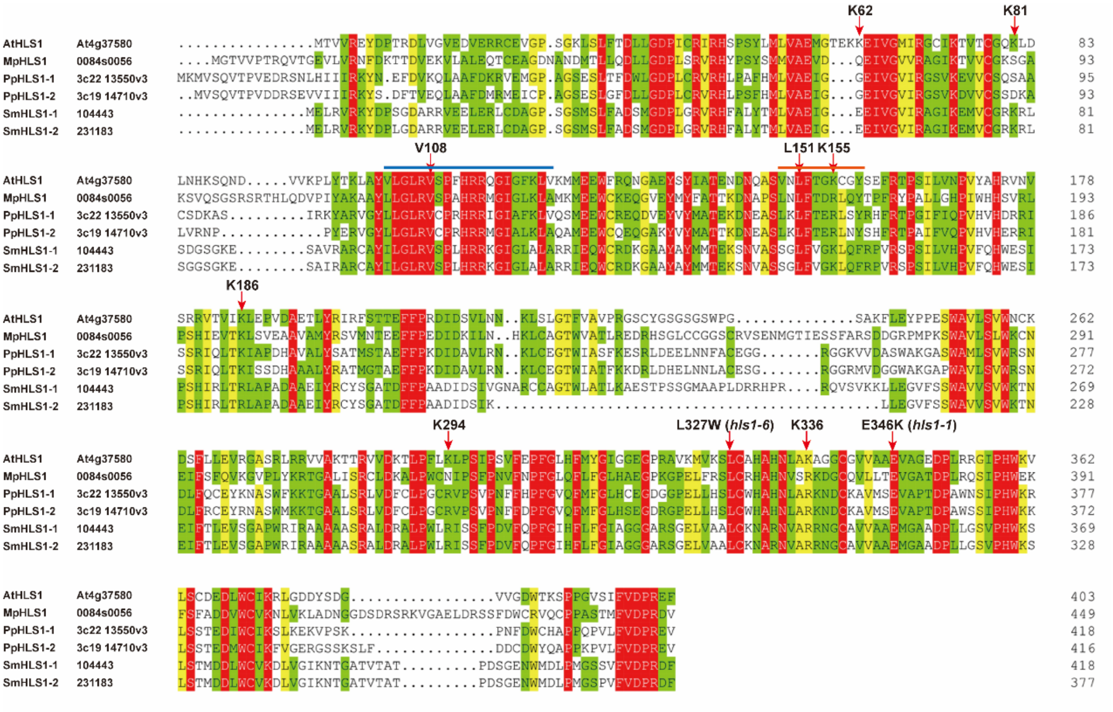

Supplementary Figure 5: **Amino acid alignment of HLS1 in different species**

The conservation levels of amino acid sequences were separately displayed in red, yellow or green. The bule line and orange line on the top of AtHLS1 sequence indicated the motif A and motif B in the conserved N-acetyltransferase domain, respectively. The short red arrows indicated key amino acid positions.

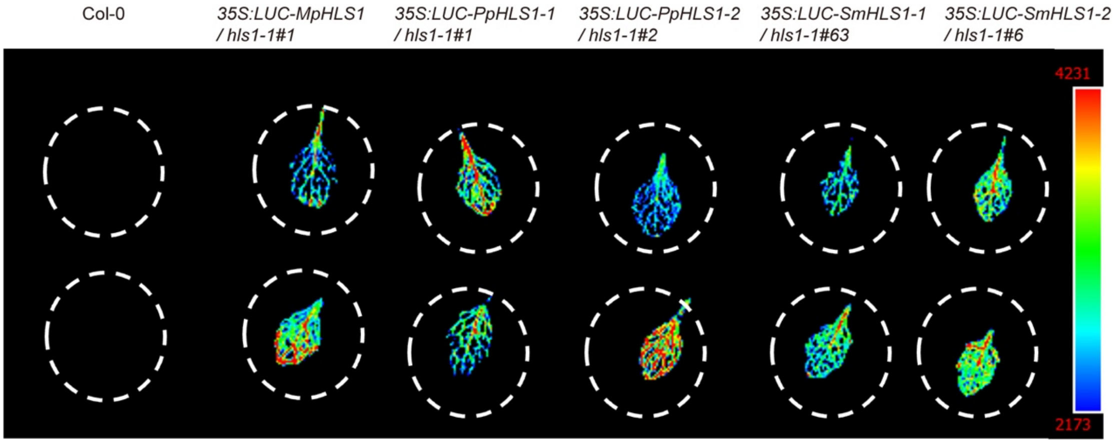

Supplementary Figure 6: **Verifying protein expressions in transgenic complementation lines**

To identify the *bona fide* overexpression lines, we detached one leaf from each T1 Basta resistant line and sprayed luciferin for luciferase activity detection. This image showed the homozygous lines obtained through this simple screening approach.

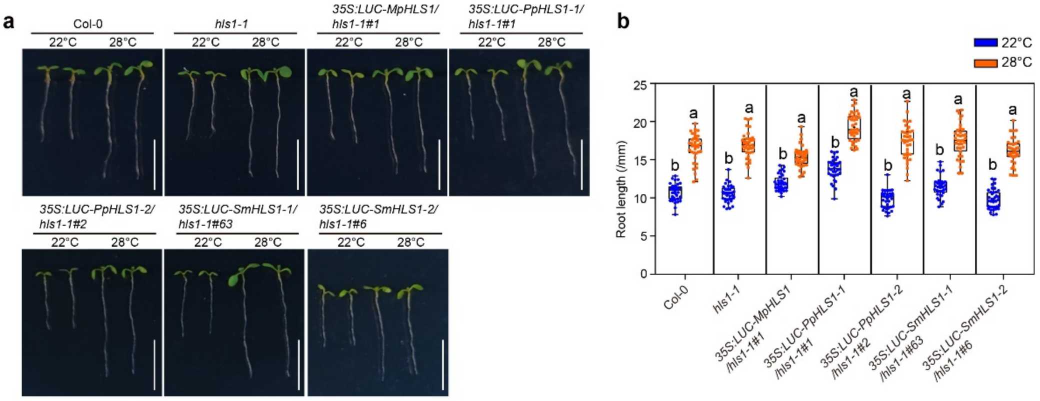

Supplementary Figure 7: **Root thermomorphogenesis phenotypes**

(a) Representative images showing the root elongation phenotypes in seedlings grown under 22 ℃ or 28 ℃. Scale bars = 1 mm.

(b) Quantification of root length. Significant differences were determined by two-way analysis of variance (ANOVA) and post hoc Tukey's test, with different lowercase letters indicating significant differences (Data are means ± SD; $n = 36$, $P < 0.05$).

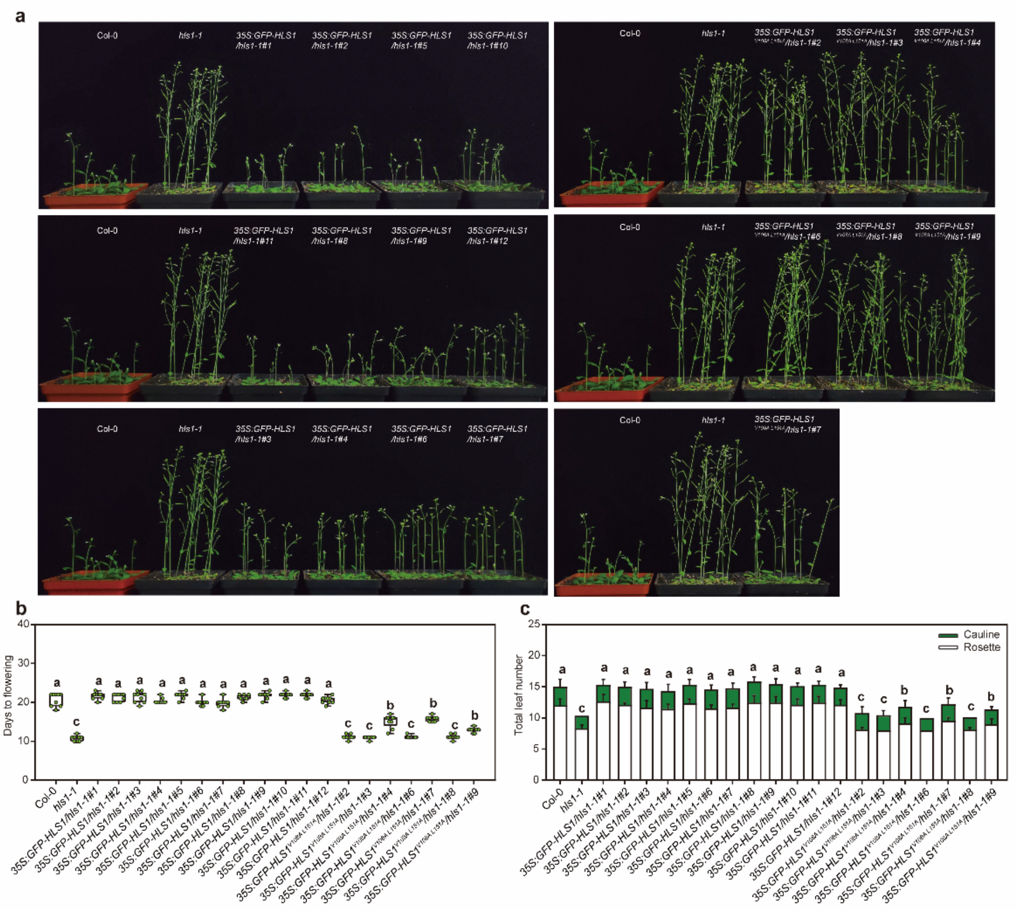

Supplementary Figure 8: **Flowering phenotypes in *GFP-AtHLS1* complementation lines**

(a) Images show the flowering time phenotypes of 27-day-old plants grown under LD conditions (16 h light/8 h dark).

(b) Statistical analysis of the days from germination to flowering under LD conditions. Significant differences were determined by one-way analysis of variance (ANOVA) and post hoc Tukey's test, with different lowercase letters indicating significant differences (Data are means ± SD; $n = 9$, $P < 0.05$).

(c) Statistical analysis the total leaf number under LD conditions. Significant differences were determined by two-way analysis of variance (ANOVA) and post hoc Tukey's test, with different lowercase letters indicating significant differences (Data are means ± SD; $n = 9$, $P < 0.05$).
