# Supplementary material for: The origin, evolution and functional divergence of HOOKLESS1 in plants
Source: Commun Biol. 2023 Apr 26;6:460. doi: 10.1038/s42003-023-04849-4 (PMC10133230; doi:10.1038/s42003-023-04849-4)
Supplement: Supplementary file 3 — Description of Additional Supplementary Files [file 42003_2023_4849_MOESM3_ESM.pdf]

Description of Additional Supplementary Files

File name: Supplementary Data 1
Description: The information of plant genomes and transcriptomes used in this study

File name: Supplementary Data 2
Description: Primers used in this study

File name: Supplementary Data 3
Description: Source data for Figure 6
