# Supplementary material for: The origin, evolution and functional divergence of HOOKLESS1 in plants
Source: Commun Biol. 2023 Apr 26;6:460. doi: 10.1038/s42003-023-04849-4 (PMC10133230; doi:10.1038/s42003-023-04849-4)
Supplement: Supplementary file 4 — Supplementary Data 1 [file 42003_2023_4849_MOESM4_ESM.docx]

**Supplementary Data 1: The information of plant genomes and transcriptomes used in this study**

*Note: the 32 representative plants were in bold.*

| **Groups** | **Species** | **Links** |
| --- | --- | --- |
| Angiosperms | ***Arabidopsis thaliana*** | http://www.angiosperms.org/ |
|  | ***Brassica rapa*** | http://www.angiosperms.org/ |
|  | ***Daucus carota*** | http://www.angiosperms.org/ |
|  | ***Oryza sativa*** | http://www.angiosperms.org/ |
|  | ***Glycine max*** | http://www.angiosperms.org/ |
|  | ***Helianthus annuus*** | http://www.angiosperms.org/ |
|  | ***Vitis vinifera*** | http://www.angiosperms.org/ |
|  | ***Olea europaea*** | http://www.angiosperms.org/ |
|  | ***Solanum lycopersicum*** | http://www.angiosperms.org/ |
|  | ***Musa acuminata*** | http://www.angiosperms.org/ |
|  | ***Setaria italica*** | http://www.angiosperms.org/ |
|  | ***Amborella trichopoda*** | http://www.angiosperms.org/ |
| Gymnosperms | ***Picea abies*** | https://www.ncbi.nlm.nih.gov/genome/11155 |
|  | ***Gnetum montanum*** | https://datadryad.org/resource/doi:10.5061/dryad.0vm37.2 |
|  | ***Ginkgo biloba*** | http://gigadb.org/dataset/100209 |
| Lycophytes | ***Salvinia cucullata*** | https://www.fernbase.org/ |
|  | ***Azolla filiculoides*** | https://www.fernbase.org/ |
|  | ***Selaginella moellendorffii*** | https://www.ncbi.nlm.nih.gov/genome/411 |
| Bryophytes | ***Sphagnum fallax*** | https://phytozome.https://phycocosm.jgi.doe.gov/phycocosm/home.doe.gov/ |
|  | ***Physcomitrella patens*** | https://www.ncbi.nlm.nih.gov/genome/383 |
|  | ***Marchantia polymorpha*** | https://www.ncbi.nlm.nih.gov/genome/3220 |
| Charophytes | ***Chara braunii*** | https://bioinformatics.psb.ugent.be/orcae/ |
|  | ***Klebsormidium nitens*** | https://www.ncbi.nlm.nih.gov/genome?term=Klebsormidium |
|  | *Bambusina borreri* | https://db.cngb.org/onekp |
|  | *Chaetosphaeridium globosum* | https://db.cngb.org/onekp |
|  | *Chara vulgaris* | https://db.cngb.org/onekp |
|  | *Closterium lunula* | https://db.cngb.org/onekp |
|  | *Coleochaete irregularis* | https://db.cngb.org/onekp |
|  | *Coleochaete scutata* | https://db.cngb.org/onekp |
|  | *Cosmarium broomei* | https://db.cngb.org/onekp |
|  | *Cosmarium granatum* | https://db.cngb.org/onekp |
|  | *Cosmarium ochthodes* | https://db.cngb.org/onekp |
|  | *Cosmarium subtumidum* | https://db.cngb.org/onekp |
|  | *Cosmarium tinctum* | https://db.cngb.org/onekp |
|  | *Cylindrocystis* sp. | https://db.cngb.org/onekp |
|  | *Cylindrocystis brebissonii* | https://db.cngb.org/onekp |
|  | *Cylindrocystis cushleckae* | https://db.cngb.org/onekp |
|  | *Desmidium aptogonum* | https://db.cngb.org/onekp |
|  | *Entransia fimbriata* | https://db.cngb.org/onekp |
|  | *Euastrum affine* | https://db.cngb.org/onekp |
|  | *Gloeochaete wittrockiana* | https://db.cngb.org/onekp |
|  | *Gonatozygon kinahanii* | https://db.cngb.org/onekp |
|  | *Klebsormidium subtile* | https://db.cngb.org/onekp |
|  | *Mesotaenium braunii* | https://db.cngb.org/onekp |
|  | *Mesotaenium caldariorum* | https://db.cngb.org/onekp |
|  | *Mesotaenium endlicherianum* | https://db.cngb.org/onekp |
|  | *Mesotaenium kramstae* | https://db.cngb.org/onekp |
|  | *Micrasterias fimbriata* | https://db.cngb.org/onekp |
|  | *Mougeotia* sp. | https://db.cngb.org/onekp |
|  | *Netrium digitus* | https://db.cngb.org/onekp |
|  | *Nucleotaenium eifelense* | https://db.cngb.org/onekp |
|  | *Onychonema laeve* | https://db.cngb.org/onekp |
|  | *Penium margaritaceum* | https://db.cngb.org/onekp |
|  | *Penium exiguum* | https://db.cngb.org/onekp |
|  | *Penium margaritaceum* | https://db.cngb.org/onekp |
|  | *Phymatodocis nordstedtiana* | https://db.cngb.org/onekp |
|  | *Planotaenium ohtanii* | https://db.cngb.org/onekp |
|  | *Pleurotaenium trabecula* | https://db.cngb.org/onekp |
|  | *Roya obtusa* | https://db.cngb.org/onekp |
|  | *Spirogyra* sp. | https://db.cngb.org/onekp |
|  | *Spirotaenia* sp. | https://db.cngb.org/onekp |
|  | *Spirotaenia minuta* | https://db.cngb.org/onekp |
|  | *Staurastrum sebaldi* | https://db.cngb.org/onekp |
|  | *Staurodesmus convergens* | https://db.cngb.org/onekp |
|  | *Staurodesmus omearii* | https://db.cngb.org/onekp |
|  | *Xanthidium antilopaeum* | https://db.cngb.org/onekp |
|  | *Zygnemopsis* sp. | https://db.cngb.org/onekp |
| Chlorophytes | *Asterochloris glomerata* | https://phycocosm.jgi.doe.gov/phycocosm/home |
|  | *Auxenochlorella protothecoides* 0710 | https://phycocosm.jgi.doe.gov/phycocosm/home |
|  | *Auxenochlorella protothecoides* UTEX 25 | https://phycocosm.jgi.doe.gov/phycocosm/home |
|  | ***Bathycoccus prasinos* RCC1105** | https://phycocosm.jgi.doe.gov/phycocosm/home |
|  | *Botryococcus braunii Showa* v2.1 | https://phycocosm.jgi.doe.gov/phycocosm/home |
|  | *Caulerpa lentillifera* | https://phycocosm.jgi.doe.gov/phycocosm/home |
|  | *Chlamydomonas eustigma* NIES-2499 | https://phycocosm.jgi.doe.gov/phycocosm/home |
|  | *Chlamydomonas incerta* SAG 7.73 | https://phycocosm.jgi.doe.gov/phycocosm/home |
|  | *Chlamydomonas priscuii* UWO241 | https://phycocosm.jgi.doe.gov/phycocosm/home |
|  | ***Chlamydomonas reinhardtii* CC-503 v5.6** | https://phycocosm.jgi.doe.gov/phycocosm/home |
|  | *Chlamydomonas schloesseri* CCAP 11/173 | https://phycocosm.jgi.doe.gov/phycocosm/home |
|  | *Chlorella* sp. A99 | https://phycocosm.jgi.doe.gov/phycocosm/home |
|  | *Chlorella sorokiniana* DOE1412 | https://phycocosm.jgi.doe.gov/phycocosm/home |
|  | *Chlorella sorokiniana* UTEX 1230 | https://phycocosm.jgi.doe.gov/phycocosm/home |
|  | *Chlorella sorokiniana* UTEX 1602 | https://phycocosm.jgi.doe.gov/phycocosm/home |
|  | *Chlorella sorokiniana* str. 1228 | https://phycocosm.jgi.doe.gov/phycocosm/home |
|  | ***Chlorella variabilis* NC64A v1.0** | https://phycocosm.jgi.doe.gov/phycocosm/home |
|  | *Chloropicon primus* CCMP1205 | https://phycocosm.jgi.doe.gov/phycocosm/home |
|  | *Chromochloris zofingiensis* SAG v5.0 | https://phycocosm.jgi.doe.gov/phycocosm/home |
|  | ***Coccomyxa subellipsoidea* C-169 v2.0** | https://phycocosm.jgi.doe.gov/phycocosm/home |
|  | *Desmodesmus armatus* UTEX B 2533 v2.0 | https://phycocosm.jgi.doe.gov/phycocosm/home |
|  | ***Dunaliella salina* CCAP19/18** | https://phycocosm.jgi.doe.gov/phycocosm/home |
|  | *Edaphochlamys debaryana* CCAP 11/70 | https://phycocosm.jgi.doe.gov/phycocosm/home |
|  | *Enallax costatus* CCAP 276/31 v1.1 | https://phycocosm.jgi.doe.gov/phycocosm/home |
|  | *Flechtneria rotunda* SEV3VF49 v1.1 | https://phycocosm.jgi.doe.gov/phycocosm/home |
|  | *Gonium pectorale* NIES-2863 | https://phycocosm.jgi.doe.gov/phycocosm/home |
|  | *Micractinium conductrix* SAG 241.80 | https://phycocosm.jgi.doe.gov/phycocosm/home |
|  | *Microglena sp.* YARC | https://phycocosm.jgi.doe.gov/phycocosm/home |
|  | *Micromonas commoda* RCC 299 | https://phycocosm.jgi.doe.gov/phycocosm/home |
|  | *Micromonas pusilla* CCMP1545 | https://phycocosm.jgi.doe.gov/phycocosm/home |
|  | *Micromonas* sp. AD1 | https://phycocosm.jgi.doe.gov/phycocosm/home |
|  | *Monoraphidium minutum* 26B-AM v1.0 | https://phycocosm.jgi.doe.gov/phycocosm/home |
|  | *Monoraphidium neglectum* SAG 48.87 | https://phycocosm.jgi.doe.gov/phycocosm/home |
|  | *Ostreococcus lucimarinus* | https://phycocosm.jgi.doe.gov/phycocosm/home |
|  | *Ostreococcus* sp. RCC809 | https://phycocosm.jgi.doe.gov/phycocosm/home |
|  | *Ostreococcus tauri* RCC1115 v1.0 | https://phycocosm.jgi.doe.gov/phycocosm/home |
|  | *Ostreococcus tauri* RCC4221 v3.0 | https://phycocosm.jgi.doe.gov/phycocosm/home |
|  | *Picochlorum renovo* | https://phycocosm.jgi.doe.gov/phycocosm/home |
|  | *Picochlorum soloecismus* DOE101 | https://phycocosm.jgi.doe.gov/phycocosm/home |
|  | *Picocystis* sp. ML | https://phycocosm.jgi.doe.gov/phycocosm/home |
|  | *Raphidocelis subcapitata* NIES-35 | https://phycocosm.jgi.doe.gov/phycocosm/home |
|  | *Scenedesmus obliquus* EN0004 v1.0 | https://phycocosm.jgi.doe.gov/phycocosm/home |
|  | *Scenedesmus obliquus* UTEX 393 | https://phycocosm.jgi.doe.gov/phycocosm/home |
|  | *Scenedesmus obliquus* UTEX 393 v2.0 | https://phycocosm.jgi.doe.gov/phycocosm/home |
|  | *Scenedesmus obliquus* UTEX B 3031 | https://phycocosm.jgi.doe.gov/phycocosm/home |
|  | *Scenedesmus obliquus* var. DOE0013 v1.0 | https://phycocosm.jgi.doe.gov/phycocosm/home |
|  | *Scenedesmus obliquus* var. UTEX 1450 | https://phycocosm.jgi.doe.gov/phycocosm/home |
|  | *Scenedesmus obliquus* var. UTEX2630 | https://phycocosm.jgi.doe.gov/phycocosm/home |
|  | *Scenedesmus* sp. NREL 46B-D3 v1.0 | https://phycocosm.jgi.doe.gov/phycocosm/home |
|  | *Symbiochloris reticulata* Africa | https://phycocosm.jgi.doe.gov/phycocosm/home |
|  | *Symbiochloris reticulata* Scotland | https://phycocosm.jgi.doe.gov/phycocosm/home |
|  | *Symbiochloris reticulata* Switzerland v1.0 | https://phycocosm.jgi.doe.gov/phycocosm/home |
|  | *Tetrabaena socialis* NIES-571 | https://phycocosm.jgi.doe.gov/phycocosm/home |
|  | *Tetradesmus deserticola* SNI-2 v1.1 | https://phycocosm.jgi.doe.gov/phycocosm/home |
|  | *Tetradesmus obliquus* UTEX 72 v1.1 | https://phycocosm.jgi.doe.gov/phycocosm/home |
|  | *Tetraselmis striata* | https://phycocosm.jgi.doe.gov/phycocosm/home |
|  | *Trebouxia sp.* A1-2 | https://phycocosm.jgi.doe.gov/phycocosm/home |
|  | ***Ulva mutabilis* Foyn** | https://phycocosm.jgi.doe.gov/phycocosm/home |
|  | *Volvox carteri* v2.1 | https://phycocosm.jgi.doe.gov/phycocosm/home |
|  | *Symbiochloris reticulata* Spain | https://phycocosm.jgi.doe.gov/phycocosm/home |
|  | *Acrosiphonia* sp. | https://db.cngb.org/onekp |
|  | *Ankistrodesmus* sp. | https://db.cngb.org/onekp |
|  | *Aphanochaete repens* | https://db.cngb.org/onekp |
|  | *Asteromonas gracilis* | https://db.cngb.org/onekp |
|  | *Bolbocoleon piliferum* | https://db.cngb.org/onekp |
|  | *Botryococcus sudeticus* | https://db.cngb.org/onekp |
|  | *Brachiomonas submarina* | https://db.cngb.org/onekp |
|  | *Carteria crucifera* | https://db.cngb.org/onekp |
|  | *Carteria obtusa* | https://db.cngb.org/onekp |
|  | *Chaetopeltis orbicularis* | https://db.cngb.org/onekp |
|  | *Chlamydomonas bilatus* | https://db.cngb.org/onekp |
|  | *Chlamydomonas cribrum* | https://db.cngb.org/onekp |
|  | *Chlamydomonas moewusii* | https://db.cngb.org/onekp |
|  | *Chlamydomonas noctigama* | https://db.cngb.org/onekp |
|  | *Chlamydomonas* sp. | https://db.cngb.org/onekp |
|  | *Chloromonas oogama* | https://db.cngb.org/onekp |
|  | *Chloromonas reticulata* | https://db.cngb.org/onekp |
|  | *Chloromonas rosae* | https://db.cngb.org/onekp |
|  | *Chloromonas subdivisa* | https://db.cngb.org/onekp |
|  | *Chloromonas tughillensis* | https://db.cngb.org/onekp |
|  | *Cladophora glomerata* | https://db.cngb.org/onekp |
|  | *Coccomyxa pringsheimii* | https://db.cngb.org/onekp |
|  | *Cylindrocapsa geminella* | https://db.cngb.org/onekp |
|  | *Cymbomonas* sp. | https://db.cngb.org/onekp |
|  | *Desmochloris halophila* | https://db.cngb.org/onekp |
|  | *Dolichomastix tenuilepis* | https://db.cngb.org/onekp |
|  | *Dunaliella tertiolecta* | https://db.cngb.org/onekp |
|  | *Dunaliella viridis* | https://db.cngb.org/onekp |
|  | *Entocladia endozoica* | https://db.cngb.org/onekp |
|  | *Eremosphaera viridis* | https://db.cngb.org/onekp |
|  | *Eudorina elegans* | https://db.cngb.org/onekp |
|  | *Fritschiella tuberosa* | https://db.cngb.org/onekp |
|  | *Geminella* sp. | https://db.cngb.org/onekp |
|  | *Golenkinia longispicula* | https://db.cngb.org/onekp |
|  | *Haematococcus pluvialis* | https://db.cngb.org/onekp |
|  | *Hafniomonas reticulata* | https://db.cngb.org/onekp |
|  | *Halochlorococcum marinum* | https://db.cngb.org/onekp |
|  | *Heterochlamydomonas inaequalis* | https://db.cngb.org/onekp |
|  | *Ignatius tetrasporus* | https://db.cngb.org/onekp |
|  | *Interfilum paradoxum* | https://db.cngb.org/onekp |
|  | *Leptosira obovata* | https://db.cngb.org/onekp |
|  | *Lobochlamys segnis* | https://db.cngb.org/onekp |
|  | *Lobomonas rostrata* | https://db.cngb.org/onekp |
|  | *Mantoniella squamata* | https://db.cngb.org/onekp |
|  | *Microspora tumidula* | https://db.cngb.org/onekp |
|  | *Monomastix opisthostigma* | https://db.cngb.org/onekp |
|  | *Nannochloris atomus* | https://db.cngb.org/onekp |
|  | *Neochlorosarcina* sp. | https://db.cngb.org/onekp |
|  | *Neodesmus pupukensis* | https://db.cngb.org/onekp |
|  | *Nephroselmis olivacea* | https://db.cngb.org/onekp |
|  | *Nephroselmis pyriformis* | https://db.cngb.org/onekp |
|  | *Ochlochaete* sp. | https://db.cngb.org/onekp |
|  | *Oedogonium cardiacum* | https://db.cngb.org/onekp |
|  | *Oedogonium foveolatum* | https://db.cngb.org/onekp |
|  | *Oltmannsiellopsis viridis* | https://db.cngb.org/onekp |
|  | *Oogamochlamys gigantea* | https://db.cngb.org/onekp |
|  | *Pandorina morum* | https://db.cngb.org/onekp |
|  | *Parachlorella kessleri* | https://db.cngb.org/onekp |
|  | *Pediastrum duplex* | https://db.cngb.org/onekp |
|  | *Percursaria percursa* | https://db.cngb.org/onekp |
|  | *Phacotus lenticularis* | https://db.cngb.org/onekp |
|  | *Pirula salina* | https://db.cngb.org/onekp |
|  | *Planophila laetevirens* | https://db.cngb.org/onekp |
|  | *Planophila terrestris* | https://db.cngb.org/onekp |
|  | *Prasiola crispa* | https://db.cngb.org/onekp |
|  | *Prototheca wickerhamii* | https://db.cngb.org/onekp |
|  | *Pseudoscourfieldia marina* | https://db.cngb.org/onekp |
|  | *Pteromonas angulosa* | https://db.cngb.org/onekp |
|  | *Pycnococcus provasolii* | https://db.cngb.org/onekp |
|  | *Pyramimonas parkeae* | https://db.cngb.org/onekp |
|  | *Scherffelia dubia* | https://db.cngb.org/onekp |
|  | *Spermatozopsis similis* | https://db.cngb.org/onekp |
|  | *Stichococcus bacillaris* | https://db.cngb.org/onekp |
|  | *Stigeoclonium helveticum* | https://db.cngb.org/onekp |
|  | *Tetraselmis chui* | https://db.cngb.org/onekp |
|  | *Tetraselmis cordiformis* | https://db.cngb.org/onekp |
|  | *Trebouxia arboricola* | https://db.cngb.org/onekp |
|  | *Trentepohlia annulata* | https://db.cngb.org/onekp |
|  | *Uronema* sp. | https://db.cngb.org/onekp |
|  | *Uronema belkae* | https://db.cngb.org/onekp |
|  | *Vitreochlamys* sp. | https://db.cngb.org/onekp |
|  | *Volvox aureus* | https://db.cngb.org/onekp |
|  | *Volvox globator* | https://db.cngb.org/onekp |
| Prasinodermophytes | *Prasinococcus capsulatus* | https://db.cngb.org/onekp |
|  | *Prasinoderma coloniale* CCMP1413 | https://phycocosm.jgi.doe.gov/phycocosm/home |
| Rhodophytes | *Galdieria sulphuraria* MS1 | https://phycocosm.jgi.doe.gov/phycocosm/home |
|  | *Galdieria sulphuraria* RT22 | https://phycocosm.jgi.doe.gov/phycocosm/home |
|  | *Galdieria sulphuraria* 074W | https://phycocosm.jgi.doe.gov/phycocosm/home |
|  | *Galdieria sulphuraria* SAG 21.92 | https://phycocosm.jgi.doe.gov/phycocosm/home |
|  | *Galdieria sulphuraria* Azora | https://phycocosm.jgi.doe.gov/phycocosm/home |
|  | *Galdieria sulphuraria* YNP5578.1 | https://phycocosm.jgi.doe.gov/phycocosm/home |
|  | *Galdieria sulphuraria* MtSh | https://phycocosm.jgi.doe.gov/phycocosm/home |
|  | *Galdieria sulphuraria* 5572 | https://phycocosm.jgi.doe.gov/phycocosm/home |
|  | *Galdieria sulphuraria* 002 | https://phycocosm.jgi.doe.gov/phycocosm/home |
|  | *Galdieria phlegrea* Soos | https://phycocosm.jgi.doe.gov/phycocosm/home |
|  | *Cyanidioschyzon merolae* strain 10D | https://phycocosm.jgi.doe.gov/phycocosm/home |
|  | ***Cyanidioschyzon merolae* Soos** | https://phycocosm.jgi.doe.gov/phycocosm/home |
|  | ***Chondrus crispus* Stackhouse** | https://phycocosm.jgi.doe.gov/phycocosm/home |
|  | *Gracilariopsis chorda isolate* SKKU-2015 | https://phycocosm.jgi.doe.gov/phycocosm/home |
|  | *Porphyra umbilicalis isolate* 4086291 | https://phycocosm.jgi.doe.gov/phycocosm/home |
|  | *Pyropia yezoensis* U-51 | https://phycocosm.jgi.doe.gov/phycocosm/home |
|  | *Ceramium kondoi* | https://db.cngb.org/onekp |
|  | *Heterosiphonia pulchra* | https://db.cngb.org/onekp |
|  | *Neosiphonia japonica* | https://db.cngb.org/onekp |
|  | *Symphyocladia latiuscula* | https://db.cngb.org/onekp |
|  | *Dumontia simplex* | https://db.cngb.org/onekp |
|  | *Gloiopeltis furcata* | https://db.cngb.org/onekp |
|  | *Chondrus crispus* | https://db.cngb.org/onekp |
|  | *Mazzaella japonica* | https://db.cngb.org/onekp |
|  | *Ahnfeltiopsis flabelliformis* | https://db.cngb.org/onekp |
|  | *Betaphycus philippinensis* | https://db.cngb.org/onekp |
|  | *Eucheuma denticulatum* | https://db.cngb.org/onekp |
|  | *Kappaphycus alvarezii* | https://db.cngb.org/onekp |
|  | *Glaucosphaera vacuolata* | https://db.cngb.org/onekp |
|  | *Gracilaria blodgettii* | https://db.cngb.org/onekp |
|  | *Gracilaria chouae* | https://db.cngb.org/onekp |
|  | *Gracilaria lemaneiformis* | https://db.cngb.org/onekp |
|  | *Gracilaria vermiculophylla* | https://db.cngb.org/onekp |
|  | *Grateloupia catenata* | https://db.cngb.org/onekp |
|  | *Grateloupia filicina* | https://db.cngb.org/onekp |
|  | *Grateloupia livida* | https://db.cngb.org/onekp |
|  | *Grateloupia turuturu* | https://db.cngb.org/onekp |
|  | *Porphyridium cruentum* | https://db.cngb.org/onekp |
|  | *Porphyridium purpureum* | https://db.cngb.org/onekp |
|  | *Rhodella violacea* | https://db.cngb.org/onekp |
|  | *Chroodactylon ornatum* | https://db.cngb.org/onekp |
| Glaucophytes | ***Cyanophora paradoxa* CCMP329** | https://phycocosm.jgi.doe.gov/phycocosm/home |
|  | *Glaucocystis cf. nostochinearum* | https://db.cngb.org/onekp |
|  | *Gloeochaete wittrockiana* | https://db.cngb.org/onekp |
|  | *Cyanoptyche gloeocystis* | https://db.cngb.org/onekp |
